# Identification and validation of a novel autoantibody biomarker panel for differential diagnosis of pancreatic ductal adenocarcinoma

**DOI:** 10.3389/fimmu.2025.1494446

**Published:** 2025-01-30

**Authors:** Metoboroghene O. Mowoe, Hisham Allam, Joshua Nqada, Marc Bernon, Karan Gandhi, Sean Burmeister, Urda Kotze, Miriam Kahn, Christo Kloppers, Suba Dharshanan, Zafirah Azween, Pamela Maimela, Paul Townsend, Eduard Jonas, Jonathan M. Blackburn

**Affiliations:** ^1^ Department of Integrative Biomedical Sciences, Division of Chemical and Systems Biology, Faculty of Health Sciences, University of Cape Town, Cape Town, South Africa; ^2^ Institute of Infectious Disease and Molecular Medicine, Faculty of Health Sciences, University of Cape Town, Cape Town, South Africa; ^3^ Surgical Gastroenterology Unit, Division of General Surgery, Groote Schuur Hospital, University of Cape Town, Cape Town, South Africa; ^4^ Recombinant Protein Facility, Sengenics Corporation, Kuala Lumpur, Malaysia; ^5^ Faculty of Health Sciences and Sports, University of Stirling, Stirling, United Kingdom

**Keywords:** pancreatic ductal adenocarcinoma, biomarker panel, diagnosis, autoantibodies, protein microarray

## Abstract

**Introduction:**

New biomarkers are urgently needed to detect pancreatic ductal adenocarcinoma (PDAC) at an earlier stage for individualized treatment strategies and to improve outcomes. Autoantibodies (AAbs) in principle make attractive biomarkers as they arise early in disease, report on disease-associated perturbations in cellular proteomes, and are static in response to other common stimuli, yet are measurable in the periphery, potentially well in advance of the onset of clinical symptoms.

**Methods:**

Here, we used high-throughput, custom cancer antigen microarrays to identify a clinically relevant autoantibody biomarker combination able to differentially detect PDAC. Specifically, we quantified the serological AAb profiles of 94 PDAC, chronic pancreatitis (CP), other pancreatic- (PC) and prostate cancers (PRC), non-ulcer dyspepsia patients (DYS), and healthy controls (HC).

**Results:**

Combinatorial ROC curve analysis on the training cohort data from the cancer antigen microarrays identified the most effective biomarker combination as CEACAM1-DPPA2-DPPA3-MAGEA4-SRC-TPBG-XAGE3 with an AUC = 85·0% (SE = 0·828, SP = 0·684). Additionally, differential expression analysis on the samples run on the iOme™ array identified 4 biomarkers (ALX1-GPA33-LIP1-SUB1) upregulated in PDAC against diseased and healthy controls. Identified AAbs were validated in silico using public immunohistochemistry datasets and experimentally using a custom PDAC protein microarray comprising the 11 optimal AAb biomarker panel. The clinical utility of the biomarker panel was tested in an independent cohort comprising 223 PDAC, PC, PRC, colorectal cancer (CRC), and HC samples. Combinatorial ROC curve analysis on the validation data identified the most effective biomarker combination to be CEACAM1-DPPA2-DPPA3-MAGEA4-SRC-TPBG-XAGE3 with an AUC = 85·0% (SE = 0·828, SP = 0·684). Subsequently, the specificity of the 11-biomarker panel was validated against other cancers (PDAC vs PC: AUC = 70·3%; PDAC vs CRC: AUC = 84·3%; PDAC vs PRC: AUC = 80·2%) and healthy controls (PDAC vs HC: AUC = 80·9%), confirming that this novel AAb biomarker panel is able to selectively detect PDAC amongst other confounding diseases.

**Conclusion:**

This AAb panel may therefore have the potential to form the basis of a novel diagnostic test for PDAC.

## 1 Introduction

Despite significant advances in cancer therapies, pancreatic cancer, of which ~90% are pancreatic ductal adenocarcinoma (PDAC), is predicted to surpass breast cancer as the 3^rd^ leading cause of global cancer deaths by 2025 (1). PDAC is characterized by an asymptomatic presentation until a late stage, with vague, intermittent symptoms and an unusual resistance to conventional therapies, resulting in a poor prognosis. Moreover, screening is made difficult by its location within the body resulting in a mere ~20% of patients diagnosed being eligible for surgical resection and a high recurrence, with a 5-y survival below 7% (2–5). The limited repertoire of treatment options is in part due to non-clinically targetable driver mutations in specific genes, coupled with a high abundance of low frequency passenger mutations, resulting in high genomic heterogeneity (6, 7).

Currently, serological carbohydrate antigen 19-9 (CA 19-9), remains the most studied and extensively used PDAC serum biomarker in the clinical setting (8) However, its use in PDAC diagnosis is limited by the fact that it is ineffacious in Lewis antigen a and b negative (Le a^-^b^-^) populations (9) whilst high levels of the biomarker are also observed in patients with various benign and malignant conditions (10). Consequently, a significant proportion of PDAC patients (up to ~30%) are misdiagnosed as other gastrointestinal diseases, such as gall bladder, gastroesophageal reflux disease, chronic pancreatitis or peptic ulcer disease (11, 12).

Classically, PDAC is immunologically cold due to a lack of inflammation in early disease, implying low T-cell recruitment. However, whilst the role of B-cells in cancer progression and treatment response remains controversial, there is increasing evidence in numerous cancers of humoral responses that are detectable in the periphery. Particularly, autoantibodies (AAbs) are known to be induced in cancers against neoepitopes arising from, amongst others, aberrantly expressed, germline-encoded fetal antigens, as well as from mutations, aberrant splicing and aberrant post-translational modifications in the cancer cell proteome. This therefore raises the enticing prospect of early cancer diagnosis through quantitation of panels of cancer antigen-specific autoantibodies that are present in serum.

In searching for novel autoantibody-autoantigen pairs that have early diagnostic potential in cancers, the cancer-testis (CT) antigens are a particularly attractive family of ca. 500 tumor-specific antigens that have highly restricted expression in normal adult somatic tissues and aberrant expression in various cancers as a result of disrupted gene regulation. Since the testis is an immune-privileged site, aberrant expression of these antigens in cancers typically triggers a spontaneous cellular (T cell) and humoral (B cell) immune response to the relevant CT antigen, the latter including the maturation of B cells against specific antigens to produce cognate autoantibodies which are detectable in the circulation. Thus, whilst PDAC is generally thought to be a poorly immunogenic disease (13, 14) due to its complex and suppressive tumor environment, it seems plausible that serological AAbs against CT antigens may be detectable in early stages of PDAC as products of immune surveillance.

Here, we identify candidate AAb biomarkers for differential detection of PDAC using a novel cancer testis antigen-focused protein microarray platform (15) and we validate the AAb panels in independent cohorts using a combination of experimental and *in silico* methods, demonstrating high sensitivity and specificity of the optimal AAb panel in discriminating PDAC patients from gastric diseases, non-PDAC pancreatic cancers, other cancers, and healthy controls.

## 2 Materials and methods

### 2.1 Study population

This study was approved by the ethics committees of the University of Cape Town Human Research [HREC 559-2018 and HREC 269/2011] and Health and Social Care of Guernsey [IJG/C5.4]), and all study participants provided written informed consent. The study population consisted of retrospective training (N=94) and cancer-specific validation (N=223) cohorts (**Table 1**).

**Table 1:** Demographics, clinical characteristics and serological carbohydrate antigen 19-9 levels according to disease cohort and healthy controls of the training and validation cohort.

Training cohort (N=94)							Validation cohort (N=223)				
Variable	PDAC n = 19	PC n = 19	CP n = 20	DYS n = 13	PRC* n = 16	HCs n = 7	PDAC n = 98	PC n = 65	CRC ** n = 16	PRC n = 20	HC n = 24
Demographic and clinical characteristics											
Age (y)	53.9 ± 9.9	53.9 ± 15.3	48.9 ± 9.7	45.9 ± 14.97	67.3 ± 7.21	31.14 ± 6.70	56.2 ± 10.1	58.4 ± 9.51	56.5 ± 11.1	68.6 ± 7.79	24.3 ± 7.49
Sex (n)											
Male	12	8	4	5	–	1	58	43	6	–	10
Female	7	11	16	8	-	6	40	22	5	-	14
Race (n)											
Black	12	20	17	6	-	3	98	65	-	-	-
Mixed	5	–	3	7	–	3	–	–	–	–	–
White	2	-	-	-	-	1	-	-	-	-	-
Serological CA 19-9											
Levels *(kU/L)*	258.0 ± 562.0	560.0 ± 1233	63.8 ± 108.0	11.7 ± 20.9	-	55.17 ± 19.94	765.1 ± 1372.6 PDAC 645.5 ± 1186.6 PC		-	708.8 ± 1370.4	64.5 ± 24.5 HC

*No CA 19-9 level data was available for PRC samples.

**There was no demographic data for 5 CRC patients.

PDAC, pancreatic ductal adenocarcinoma; PC, other pancreatic cancers; CP, chronic pancreatitis; DYS, non-ulcer dyspepsia; PRC, prostate cancer; CRC, colorectal cancer; HC, healthy controls.

#### 2.1.1 Training cohort

Blood and tissue samples were collected from 94 patients diagnosed using the international classification of diseases for oncology [ICD-0]). Blood from 19 PDAC (Stage II-III), 20 chronic pancreatitis (CP), 1 other pancreatic cancer (PC; defined as non-PDAC; Stage II-III), and 13 dyspeptic ulcer (DYS) patients, as well as 7 healthy controls (HCs) were collected from Groote Schuur hospital. Tumor and adjacent “normal” tissue were also collected from ten of the PDAC patients. Additionally, 18 PC and 16 prostate cancer (PRC) samples were provided by the University of Witwatersrand National Cancer Registry and Manchester University, UK, respectively (**Table 1**).

#### 2.1.2 Cancer-specific validation cohort

Banked serum samples from PDAC (n=98; Stage II-III) and PC (n=65; Stage II-III) patients provided from the University of Witwatersrand National Cancer Registry, together with banked sera from patients with other cancers (PRC, n=20; colorectal cancer (CRC), n=16), were used to evaluate marker specificity. A total of 24 HCs (defined as persons without cancer or other gastrointestinal diseases) were also included in the validation cohort.

### 2.2 Preparation of blood and tissue samples

Blood and corresponding tumor tissue from PDAC, CP and DYS patients receiving standard of care at Groote Schuur hospital (GSH), Cape Town, were collected prospectively with written informed consent at the point of resective surgery/biopsy.

Serum was isolated from the blood by centrifugation at 1500 g × 15 min (22 °C), the supernatant was centrifuged again at 3500 g × 15 (22 °C) min to remove platelets, and then placed in clean 1·5 mL polypropylene tubes and stored at -80 °C until ready for use.

The tissue samples were collected immediately after resective surgery, sectioned into aliquots, and washed in 20 µg/ml streptomycin in PBS three times to remove any contaminants and debris. Subsequently, these were stored in organoid storage buffer (90% FBS, 10% DMSO), the cryovials were placed in a Mr. Frosty, to prevent cell damage, and placed in a -80 °C freezer overnight before being stored directly at -80 °C until ready for analysis.

### 2.3 Carbohydrate antigen 19-9 enzyme-linked immunosorbent assay

Serological levels of CA 19-9 in PDAC, CP, and DYS patients, as well as HCs from the training cohort (N= 77) were measured in duplicate wells using a human CA 19-9 enzyme-linked immunosorbent assay (Cat #DE5069; Demeditec) and quantified in k/UL.

### 2.4 Fabrication of CT100+ microarray

CT100+ microarrays comprising 113 CT- or tumor-associated antigens (**Supplementary Tables 1A, B**) were fabricated in-house as previously described (16, 17) and used to identify AAbs that were differentially present in PDAC patient sera relative to controls. (**Supplementary Figure S1A**). Briefly, antigen lysates, diluted two-fold with 40% sucrose, were printed in a 4-plex format (i.e., 4 replica arrays per slide) on streptavidin-coated hydrogel microarray substrates, and within each array/plex, the antigens were printed in technical triplicate (**Supplementary Figure S1B**). Subsequently, slides were incubated in blocking buffer (**Supplementary Table S2**) for 1 h at room temperature (RT; 22 °C). Slides were then washed in 3 × 5 min in PBST (0.1% Tween^®^-20), rinsed 1 × 5 min in ddH_2_O, and dried by centrifugation (1400 RCF for 4 min, 24 °C). Dried slides were stored in light-protected slide holders at 4 °C until ready for assay.

For each printed slide batch, successful immobilization of *in situ* purified biotinylated proteins from lysates onto the microarray substrate was confirmed using an anti-c-Myc antibody (Cat# C6594-5ML; Sigma Aldrich) assay (**Supplementary Figures S2A–D**).

### 2.5 Serological assays

CT100+ microarray slides were assayed in 4-plex, multi-well hybridization cassettes (ArrayIt). iOme v5 slides (1622 antigens; Sengenics) were assayed in single-plex, as per manufacturer’s instructions. All incubation, wash and rinse steps on both array types were performed on an orbital shaker at 100 rpm, with minimum light exposure. Serum samples (1:800; Serum: PBST; v:v) were incubated for 1 h at RT, followed by a 3 × 5 min wash in PBST and 1 × 5 min rinse step in ddH_2_0. Slides were then incubated with 20 μg/ml of Cy5-labelled anti-human IgG detection antibody (Cat # A21445; ThermoFischer Scientific) diluted in PBST for 30 min, then washed, and rinsed as above. Rinsed slides were placed in clean 50 mL polypropylene tubes and dried by centrifugation at 1400 RCF for 4 min at 24 °C.

Slides were then scanned using an InnoScan 710 AL (Innopsys, France) microarray reader, essentially as previously described. Scanning parameters are given in **Supplementary Table S3**. The resulting scans were saved as TIFF files (used for data extraction downstream) and also as JPEG files (used for data presentation and visualization during quality control steps). Data was extracted in Genepix (version 7; Molecular Devices) and processed using the Pro-MAP pipeline [25] to provide background subtracted, normalized net intensity values for each antigen-specific autoantibody in each sample.

### 2.6 Validation of identified biomarkers

#### 2.6.1 *In silico* verification of autoantigen targets

To determine the spatial localization and disease specificity of the autoantigen targets of the identified AAb biomarkers, IHC data on the presence and spatial localization of each autoantigen was retrieved from the Human Protein Atlas. Additionally, searches were performed on proteins for which no data was available in the Human Protein Atlas database using PubMed, Science Direct, and Google Scholar. The broad search concepts of the <antigen of interest>, <pancreatic cancer>, <immunohistochemistry>, and <tumor tissue> were combined into search statements specific to each database queried. An initial screening of titles and abstracts excluded letters, editorials, posters, and opinion pieces. Full text articles were screened and included if the study addressed immunohistochemistry analysis of human PDAC tumor tissue or the presence of the relevant autoantigen in PDAC tumor via tissue analysis.

#### 2.6.2 Experimental validation of PDAC selective autoantibody panels in an independent cohort through use of a custom pancreatic ductal adenocarcinoma microarray

The corresponding antigens of the 11 most discriminatory AAbs identified in the training cohort were re-expressed and used to fabricate a custom PDAC microarray. Briefly, 24-well deep plates were seeded with SuperSf9-3 cells (Oxford Expression Technologies) at 6 × 106 cells/well then infected with 50 µl of recombinant baculoviruses generated as previously described (18). The plates were incubated with agitation at 27 °C for 72 h. Subsequently, cells were harvested by centrifugation for 5 min and washed three times with 3ml of PBS buffer between each centrifugation. The pellets were then resuspended in 400 µl of freezing buffer (25mM HEPES, 50 mM KCl, pH 7·5).

Antigen expression and *in vivo* biotinylation was confirmed via western blot analysis using a streptavidin–HRP conjugate probe (GE Healthcare) (**Supplementary Table S5**, **Supplementary Figures S3, S4**). These antigens were then printed as before, except in a 16-plex format; within each plex, each antigen was printed in triplicate. Subsequently, the printed slides were assayed and scanned as described above for the CT100+ arrays.

### 2.7 Statistical analysis

For the CA 19-9 data, the frequency of Le a^-^b^-^ individuals was determined by computing a frequency distribution table in R. To determine if the data was normally distributed for subsequent analyses, a Shapiro-Wilks test was run. Non-normal data was Log or Tukey’s transformed to normalize distribution. The utility of CA 19-9 levels in distinguishing PDAC from other pancreatic diseases was then determined using receiver-operating-characteristic (ROC) curve analysis, reporting area under the ROC curve (AUC) and 95% confidence intervals. The sensitivity and specificity were derived using non-parametric re-sampling with the percentile method (2000 stratified bootstrap replicates) (19). To determine if CA 19-9 levels differed significantly across PDAC, diseased- and healthy controls, we ran a multivariate analysis of variance (MANOVA). Subsequently, we computed a TukeyHSD multiple pairwise comparison to determine if the mean difference between specific pairs was different.

For microarray analyses of the training and validation cohorts, pre-processing steps were conducted using the Pro-MAP pipeline (20). Prior to analyses, a power analysis for ROC curve analyses was run in R to determine the effect size of potential results, based on the sample sizes utilized. For the CT100+ dataset, ROC analyses were run to identify the top ten biomarkers differentiating PDAC from the diseased controls. Subsequently, a combinatorial analysis of these biomarkers was conducted using the *CombiROC* package in R (21). Briefly, a generalized linear model was applied to each combination and the resulting predictions were used to calculate ROC curves and their corresponding coordinates. The biomarker combinations were ranked based on their AUC values and the top combination was selected for the PDAC custom chip.

For the samples run on the custom PDAC microarray, a combinatorial analysis of the identified biomarker panel was run, comparing PDAC to other cancers and HCs to determine its clinical utility.

Gene ontology and subsequent Reactome pathway analyses of differentially expressed genes that made up the biomarker panel were conducted using the *clusterProfiler* (22) and *ReactomePA* (23)packages in R, respectively.

All analyses were run in R (v 4·0) and all plots were created using the *ggplot2* (24) package.

## 3 Results

### 3.1 Power calculations

Power calculations for the training cohort (N = 94) on the CT100+ array showed that, for a ROC analysis test of n_cases_ = 20 and n_controls_ = 74, an AUC = 0.80, and a significance level = 0.01, we had a power = 0.94. However, for the iOMe array, for which we were availed fewer arrays, a fold change analysis test of n_groups_ = 5 and n_n_per_group_ = 10, an effect size = 0.5, and a significance level = 0.05, we had a power = 0.77. Similarly, power analysis for the validation cohort (N = 223) showed that, for the ROC analysis test of n_cases_ = 98 and n_controls_ = 125, we had a power = 0.99. Furthermore, MANOVAs showed no significant effects of age, gender, and race on CA 19-9 or AAb profiles in disease and healthy controls of the training and validation cohorts (p > 0.05). Thus, our analyses were confirmed to be statistically powered.

### 3.2 Carbohydrate antigen 19-9 enzyme-linked immunosorbent assay

**Figure 1:**
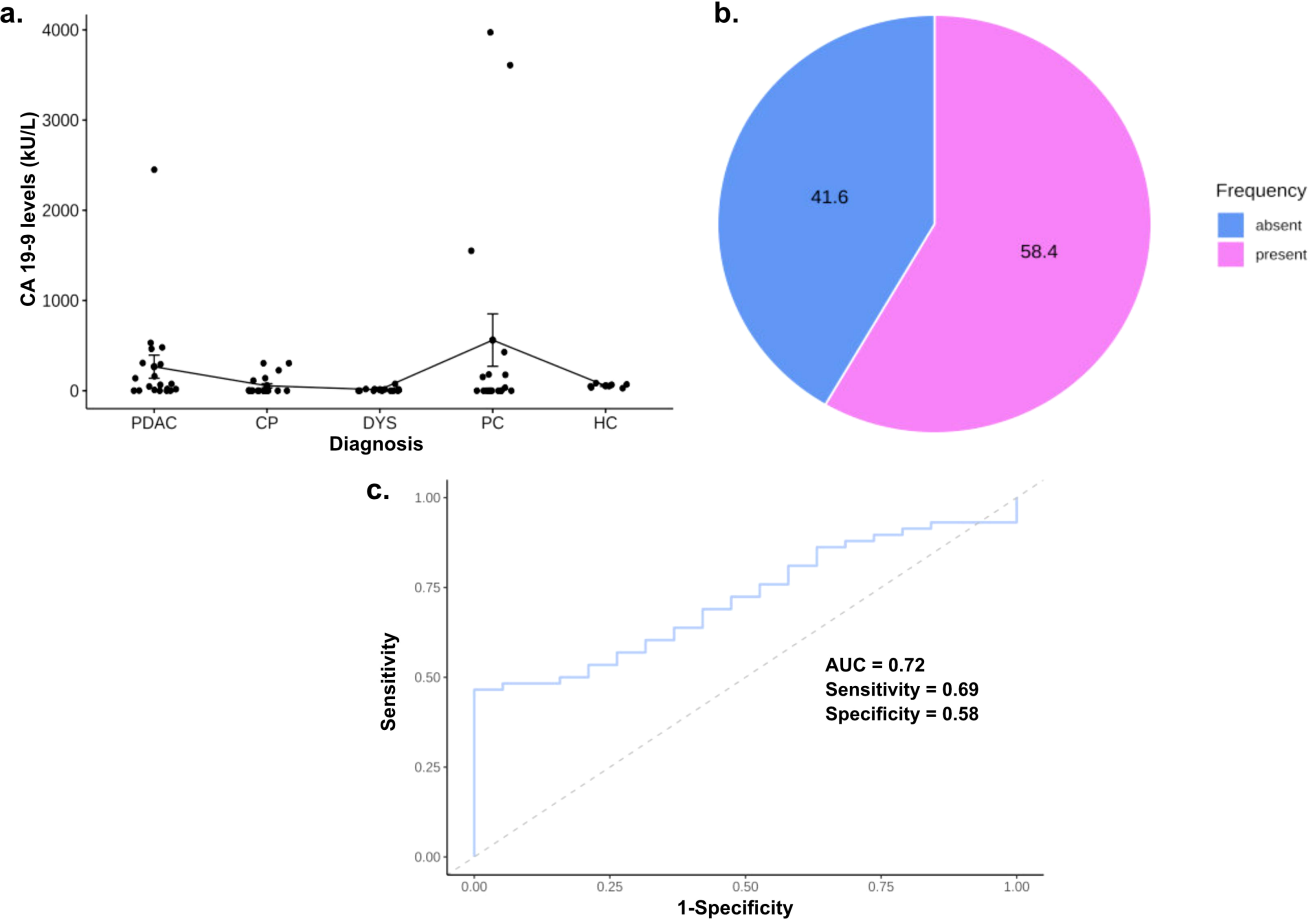
Carbohydrate antigen 19-9 (CA19-9) as a biomarker for pancreatic ductal adenocarcinoma. **(A)** Proportion of asymptomatic serological carbohydrate antigen 19-9 population in training cohort; **(B)** Boxplot portraying CA 19-9 levels in PDAC and disease controls; **(C)** ROC curves for patients with PDAC compared to all disease controls.

We generated CA 19-9 data for 77 of the 94 samples in the training cohort and found that average CA 19_9 levels were higher in PDAC than other disease and healthy controls, except PC (**Table 1**). Nevertheless, mean CA 19-9 levels did not differ significantly across PDAC, diseased- and healthy controls (F = 1.95, p = 0.11; **Figure 1A**). In line with the literature, we found that close to half of the individuals (32/77, 41.6%; **Figure 1B**) had no measurable CA 19-9 by ELISA. ROC curve analysis produced an AUC of 0.716 for PDAC patients compared to the other diseased and HCs; a cut-off of 42.19 kU/L had the optimal sensitivity and specificity of 69.0% and 57.9%, respectively (**Figure 1C**).

### 3.3 Autoantibody biomarker identification and validation

#### 3.3.1 Combined autoantibody and CA19-9 analyses of training cohort

**Figure 2:**
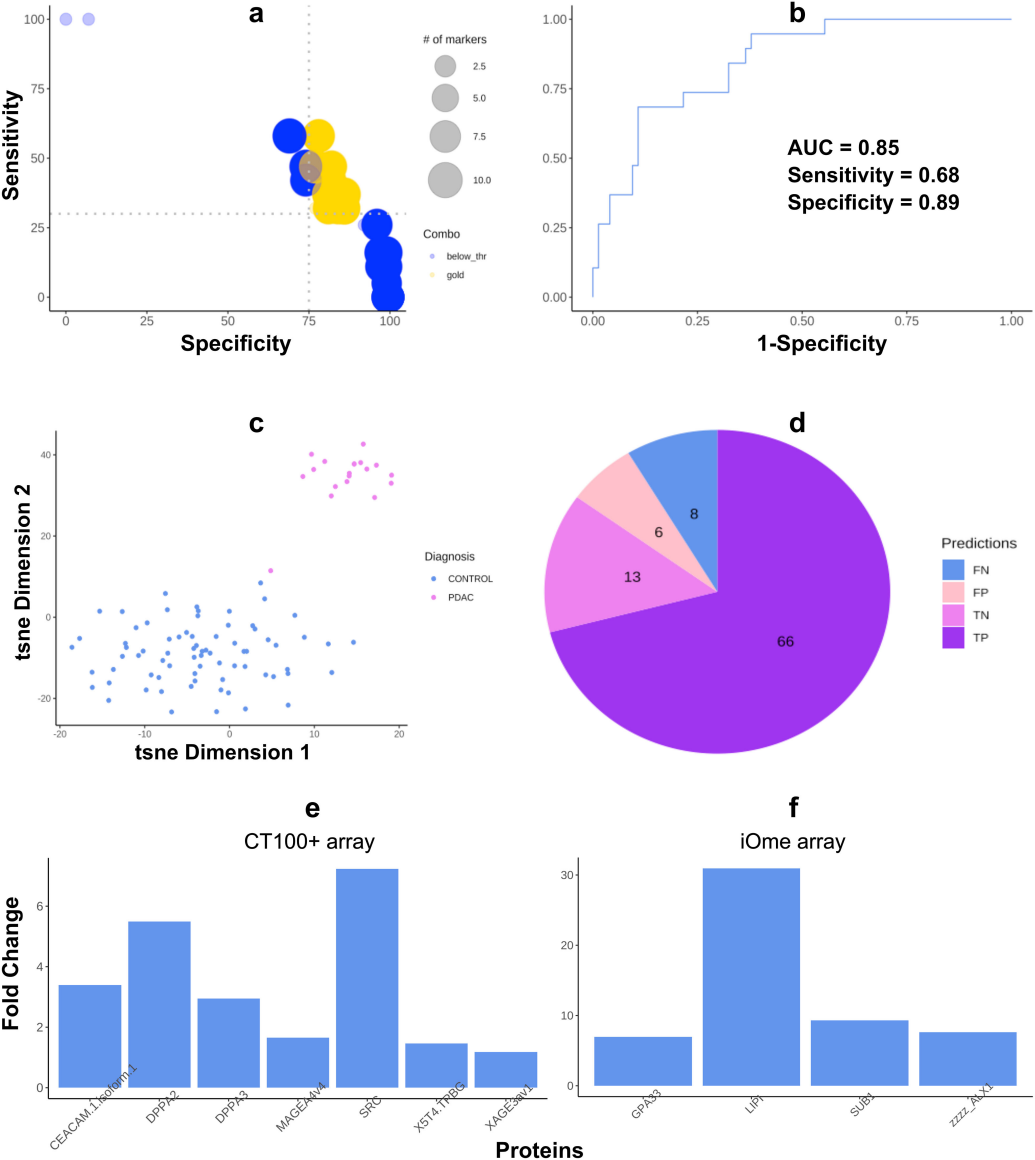
Autoantigen biomarker combination most effective for pancreatic ductal adenocarcinoma (PDAC) diagnosis. **(A)** Bubble chart discriminating between combinations not passing the user-defined SE (35) and SP (75) cut-offs (blue bubbles) and *“gold” combinations* passing them (yellow bubbles). **(B)** ROC curve of the diagnostic combination consisting of seven autoantigens; **(C)** t-SNE plots of seven autoantigens that most effectively distinguish PDAC patients from chronic pancreatitis, non-ulcer dyspepsia, prostate cancer, and healthy controls; **(D)** pie chart of case and control predictions made by the biomarker combination (FN = False negatives, FP = False positives, TN = True negatives, TP = True positives). **(E)** Barplot showing the fold change of expression intensity of PDAC versus diseased and healthy control samples on the CT100+ array. **(F)** Barplot showing the fold change of expression intensity ofautoantibodies upregulated in PDAC versus all diseased and healthy control samples on the iOme array: PDAC vs CP; PDAC vs PC; PDAC vs DYS; & PDAC vs HC comparisons.

Sera from the training cohort (N=94) were assayed on custom CT100+ microarrays and bound antigen-specific IgG autoantibodies were detected and quantified using a Cy5-labelled anti-human IgG secondary antibody. Extracted microarray data were pre-processed using Pro-MAP (20). Briefly, spots below the noise threshold of intensities <2SD were filtered out then data was normexp background corrected, normalized using the cyclic loess method and array weights were calculated following which data was consolidated into mean intensities with columns and rows representing arrays and proteins in preparation for analyses. Subsequently, a combinatorial ROC curve analysis was run on the CT100+ microarray data to identify the most effective biomarker combination on the training cohort. Based on the rankings, a biomarker combination consisting of 7 biomarkers (CEACAM1-DPPA2-DPPA3-MAGEA4-SRC-TPBG-XAGE3) had the highest AUC = 0.850, with sensitivity and specificity values of 0.828 and 0.684 respectively (**Figures 2A–C, E**). This biomarker panel was thus able to accurately predict 83% of cases (**Figure 2D**). The inclusion of CA19-9 ELISA data together with the AAb biomarker panel decreased the sensitivity slightly (0.789) but significantly increased the specificity (0.794) of the panel, yielding an AUC of 0.841.

Pooled serum samples (n = 5 per pool; 2 pools per PDAC and disease control groups; 1 pool for HCs) were assayed on iOme v5 arrays, bound antigen-specific IgG autoantibodies were detected and quantified as before, and extracted microarray was again pre-processed using Pro-MAP. Due to the smaller sample size in the iOme data, a log fold change analysis was carried out using limma and 14 differentially expressed proteins (**Figure 2F**), distinguishing PDAC from each diseased- and healthy control group, were identified. Of these, one biomarker (LIPI) had been previously identified as one of the top ten proteins following ROC analysis of CT100+ proteins and three (SUB1, ALX1, GPA33) were upregulated in PDAC compared to the other diseased cohorts and healthy controls. Thus, these four proteins were therefore also included in downstream validation studies.

#### 3.3.2 *In silico* immunohistochemistry analyses of candidate biomarkers

The experimental identification of cancer biomarkers does not guarantee their translation into the clinical setting. For clinical use, candidate biomarkers therefore need to be validated in independent cohorts, preferably including orthogonal experimental methods. Here the identified biomarkers were initially verified *in silico* through query of immunohistochemical (IHC) databases prior to experimental validation in an independent cohort using a custom PDAC protein microarray.

For *in silico* analysis of candidate biomarkers, we used the Human Protein Atlas and available literature to collect IHC data on the expression and spatial localization of the autoantigen targets of the identified autoantibody biomarkers (**Table 2**). Antigen expression data for PDAC tumor tissue was available and collected for 8 of the 11 proteins in the proposed PDAC biomarker panel. No IHC data could be extracted on DPPA3, GPA33, or LIPI protein expression in PDAC tissue. However, we found strong IHC expression of DPPA2, MAGEA4, SRC, TPBG, ALX1, and SRC and moderate expression of XAGE3 and CEACAM1. Interestingly, RNA expression in tumor tissue was only evident for CEACAM1, MAGEA4, SRC, TPBG, GPA33, and SUB1, with the highest and lowest expression for SRC and MAGEA4, respectively.

**Table 2:** Immunohistochemistry data on the expression and spatial localization of identified autoantibodies in pancreatic cancer.

Protein	Protein Expression in normal pancreatic tissue	IHC Expression (Y/N) and Intensity in pancreatic tumour tissue	Localization in tumour tissue	RNA expression levels in tumour tissue (Average FPKM)
CEACAM 1	Not detected	Y – Moderate to negative	Cytoplasmic/membranous	15.0
DPPA2	Not detected	Y –Strong to moderate	Nuclear	0.0
DPPA3	Not detected	Not applicable	Not applicable	0.0
MAGEA4	Not detected	Y – Strong to negative	Nuclear, Cytoplasmic/membranous/nuclear	0.1
SRC	Not detected	Y - Strong to weak	Cytoplasmic/membranous, Cytoplasmic/membranous/nucleus	21.3
TPBG	Detected	Y – Strong to moderate	Cytoplasmic/membranous	6.5
XAGE3	Not detected	Y – Moderate to negative	Cytoplasmic/membranous	0.0
ALX1	Detected	Y – Strong to negative	Nuclear, Cytoplasmic/membranous, Cytoplasmic/membranous/nuclear	0.0
GPA33	Not detected	Not applicable	Not applicable	2.8
LIPI	Not detected	Not applicable	Not applicable	0.0
SUB1	Detected	Y – Strong to weak	Nuclear	12.1

#### 3.3.3 Experimental analysis of an independent validation cohort using a custom pancreatic ductal adenocarcinoma microarray

**Figure 3:**
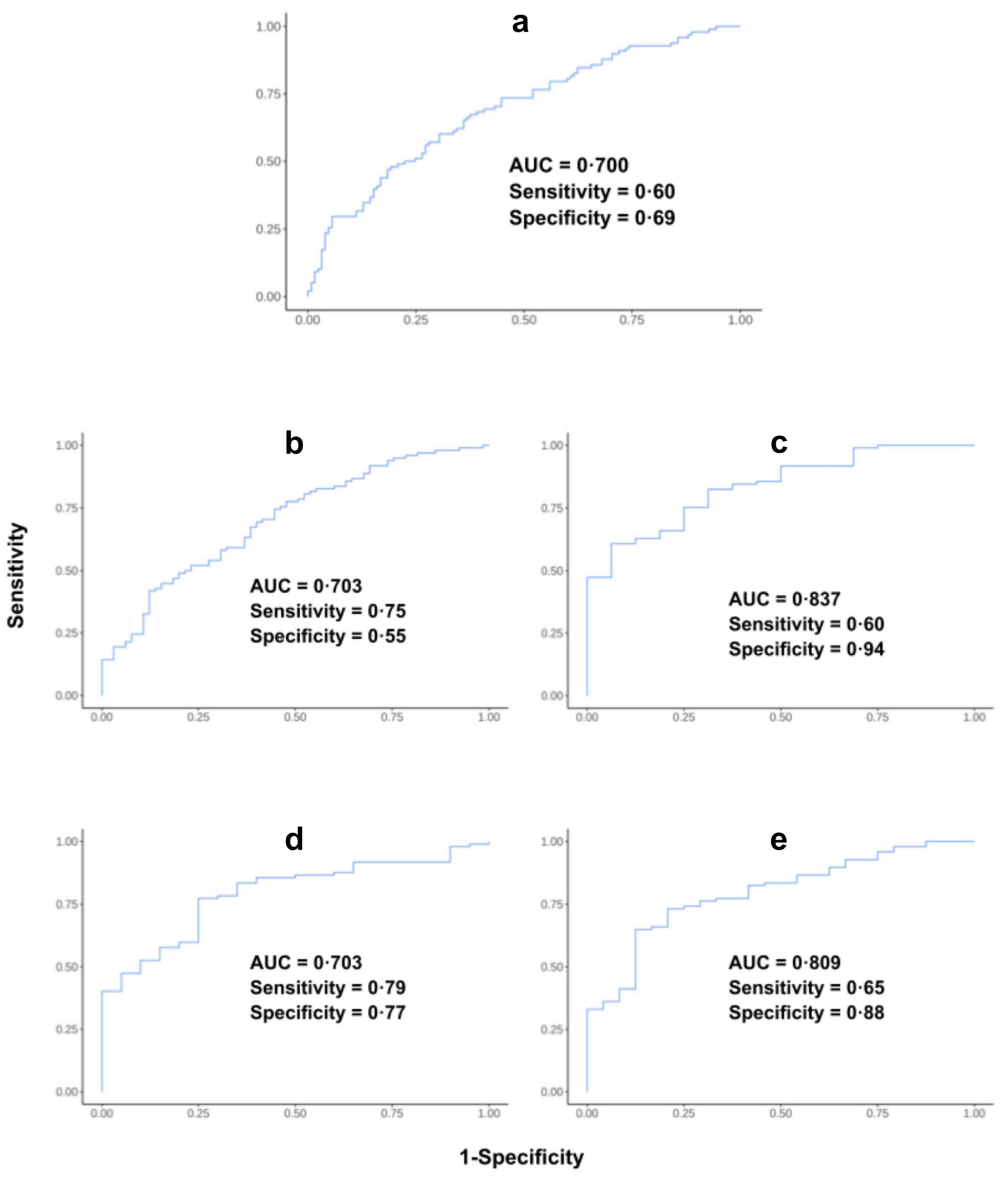
ROC curve analysis of validation cohort samples run on the custom pancreatic ductal adenocarcinoma (PDAC) array. ROC analysis curve of **(A)** PDAC versus all diseased and healthy controls; **(B)** PDAC versus other pancreatic cancers; **(C)** PDAC versus colorectal cancers; **(D)** PDAC versus prostate cancers; and **(E)** PDAC versus healthy controls.

Following *in silico* verification of expression profiles of the target autoantigens, a custom PDAC microarray consisting of the top 11 proteins identified in the training cohort (CEACAM1; DPPA2; DPPA3; MAGEA4; SRC; TPBG; XAGE3; ALX1; GPA33; LIPI; SUB1) was constructed. To determine the discriminatory power and disease specificity of our identified biomarker panel in relation to other cancers as well as HCs, serological assays were performed on an independent validation cohort (N=223) using the custom PDAC array and a combinatorial ROC curve analysis was run. Overall, in the cancer cohort, our biomarker panel had an AUC of 70% (SE = 0.60, SP = 0.69) (**Figure 3A**). Our panel was found to be least effective when comparing PDAC with PCs (AUC = 70.3, SE = 0.745, SP = 0.554; **Figure 3B**), as expected, but was more effective when distinguishing PDAC from CRC (AUC = 84.3%, SE = 0.60, SP = 0.94), PRC (AUC = 80.2%, SE = 0.79, SP = 0.77), and HC (AUC = 80.9%, SE = 0.65, SP = 0.88) (**Figures 3C–E**). With the addition of patient CA 19-9 to the panel, the panel was much more effective when distinguishing PDAC from HC (AUC = 89.2%, SE = 0.72, SP = 1). However, the addition of CA 19-9 decreased biomarker panel performance when distinguishing PDAC from all diseased and healthy controls (AUC = 62.5%, SE = 0.63, SP = 0.60), PCs (AUC = 60.8%, SE = 0.30, SP = 0.91) and PRC (AUC = 67.5%, SE = 0.61, SP = 0.70). Unfortunately, we were unable to collect CA 19-9 data for the CRC patients (**Supplementary Figure S5**).

## 4 Discussion

Pancreatic cancer is one of the most challenging cancers to detect early; thus, incidence closely parallels mortality. Currently, blood CA 19-9, imaging tests, and endoscopic ultrasounds are the most commonly used tools for PDAC detection with little improvement to diagnosis and prognosis. The advantages of exploring potential AAb biomarkers in liquid biopsies are the minimally invasive sample extraction, their high specificity, and reproducible results. Additionally, the presence of AAbs directed against cancer antigens may occur months to years prior to the onset of symptoms, thereby offering the possibility of early, pre-symptomatic diagnosis.

Based on combinatorial ROC and differential analyses of PDAC, diseased and HC samples run on the CT100+ and iOme arrays, we identified an 11 AAb biomarker panel that could effectively differentially detect PDAC and distinguish it from other related disease controls. Of the 11 biomarkers identified, ten of the corresponding antigenic targets had been previously identified in PC based on the literature, the Human Protein Atlas or both. However, to our knowledge, their autoantigenicity in PDAC was previously unknown, making their identification here as candidate AAb-based diagnostic biomarkers of PDAC both novel and potentially clinically important.

Though the sample size of the training cohort was small (n =94), with 19 PDAC samples, power analyses indicated this was sufficient for biomarker identification. Moreover, the combinatorial analyses we employed leveraged the potential synergistic effects of multiple markers, thereby minimizing the reliance on any single biomarkers performance and mitigating limitations posed by our small cohort size. Nevertheless, to address concerns about overfitting or selection bias that could arise from using a small training cohort in combinatorial ROC analyses, we tested the identified biomarker combinations in a larger, independent, validation cohort.

Using a custom array based on the biomarkers identified in the training cohort we ran assays on a larger (n =223) validation cohort to identify our final biomarker panel. The good performance of our biomarker panel on a larger, more diverse cohort highlights its generalizability and stability. The identified biomarker panel was effective against diseased (PC: AUC = 70.3%; CRC: AUC = 84.3%; PRC: AUC = 80.2%) and healthy controls (AUC = 80.9%). Our exploratory analyses suggested that the diagnostic specificity of our panel may be further improved with the addition of CA19-9; thus, we tested this on our validation cohort. We found that though CA 19-9 enhanced the performance of our biomarker panel when distinguishing PDAC from healthy controls, its discriminatory power was reduced when comparing PDAC with other pancreatic cancers and prostate cancer (**Supplementary Figure S5**). This is unsurprising as it may reflect the overlapping expression of CA 19-9 in various malignancies and pancreatic diseases, which limits its specificity in distinguishing PDAC from other cancers and pancreatic diseases.

The lower AUC against other pancreatic cancers (PC) is expected as these diseases are the most similar to PDAC. The overall variability across disease pairs results from the unique pathophysiological mechanisms and biomarker expression patterns of the different diseases. Notwithstanding, the AUC for healthy controls and other cancers showed a good overall performance. However, its clinical utility may depend on the specific prevalence of each disease in the population being tested. This underscores the importance for disease-specific validation and refinement of biomarker panels and may suggest the expansion or further refinement of our panel to improve the performance for diseases with a lower AUC. Thus, for clinical use, the diagnostic power of our biomarker panel should be explored in a larger independent PDAC cohort including individuals which carry an increased risk of developing PDAC as well as in other more closely related cancers, in order to confirm its tissue specificity. It would also be interesting to explore the utility of our panel on a European and Asian cohorts to fully realize the generalizability of our panel on a global scale.

**Figure 4:**
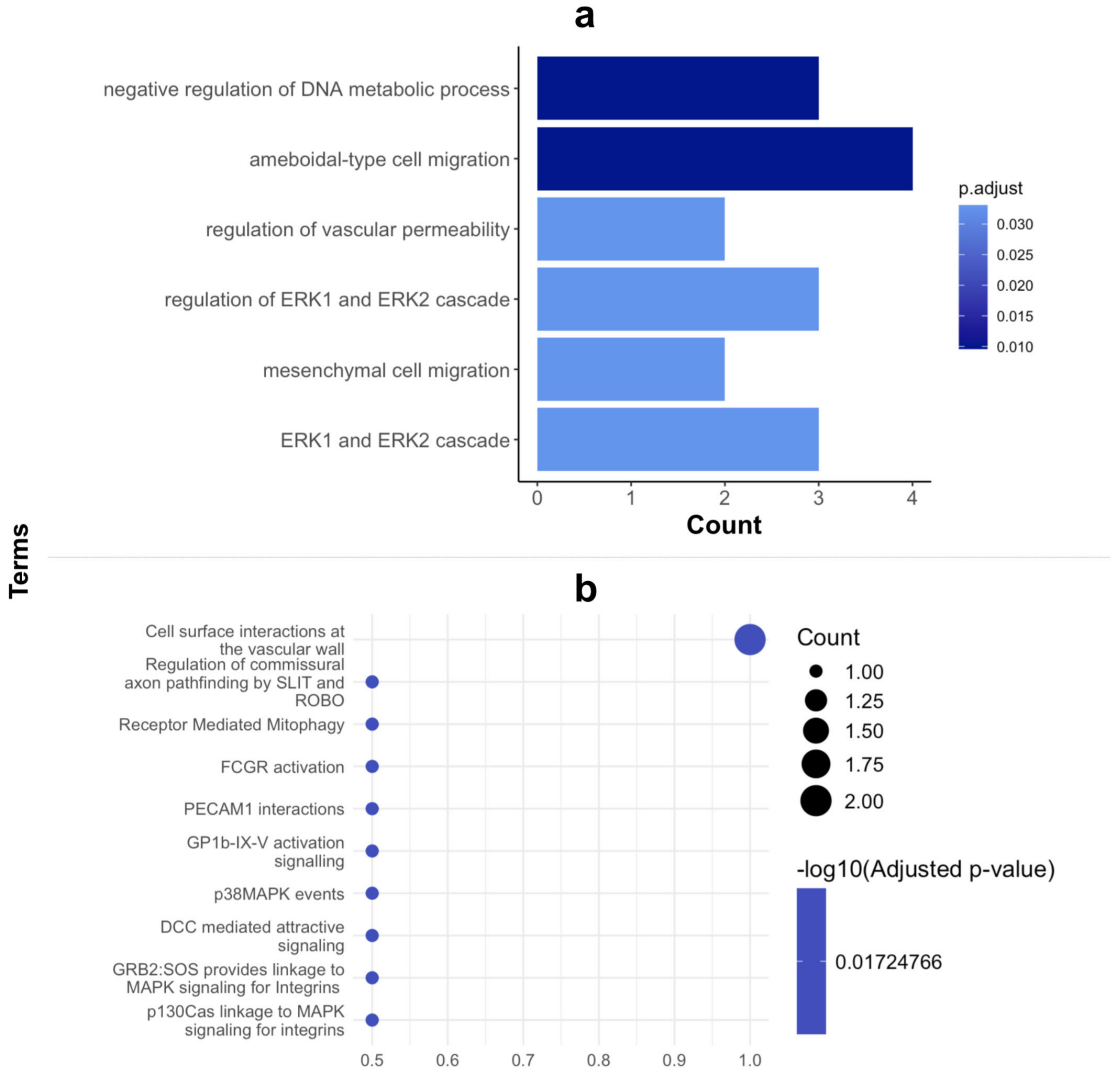
GO Enrichment and PATHWAY analysis of proteins in biomarker panel. **(A)** Cluster profiler GO enrichment analysis of biological processes and **(B)** Reactome PATHWAY analysis of biomarker proteins.

GO enrichment analysis of our candidate biomarker panel highlighted the roles of the corresponding autoantigens in the negative regulation of metabolism, ameboidal-type cell migration, regulation of vascular permeability, and the ERK1 and ERK2 cascade (**Figure 4A**).

The negative regulation of DNA metabolism or metabolic reprogramming is a well-recognized hallmark of cancers (25), since the metabolic pathway activity of lipids, glucose, amino acids, and fatty acids (26–28) change during tumorigenesis and cancer progression to fulfil the energy biosynthetic needs of their uncontrolled proliferation (29). In PDAC, this is particularly pronounced due to the high metabolic rate of the cancer. Increased synthesis and storage of lipids contribute to cell membrane biogenesis, energy storage, and signaling all crucial for tumor progression and metastasis. Moreover, PDAC cells shift towards aerobic glycolysis (i.e. the Warburg effect) becoming reliant on glucose fermentation for energy production. This along with fatty acid oxidation produces ATP as well as intermediates necessary for biosynthesis, such as amino acids (30, 31). Subsequently, increased amino acid metabolism and uptake, particularly of glutamine, supports tumor growth and survival (32).

Ameboidal migration, likely induced by cytokines and mechanical cues, aids in the migration, dissemination and survival of cancer cells enabling their proliferation. Thus, cancer cells, including PDAC cells can modulate their migration by adopting an ameboid migration style, which allows for more flexibility when navigating ECM and blood vessel walls and may partly explain the faster migration of PDAC cells (33). Increased vascular permeability is indispensable for cancer metastasis and is highly correlated with the endothelial extravasation of tumor cells as they require movement through the endothelial cell barrier to migrate (34, 35). Pancreatic ductal adenocarcinoma is defined in part by its aggressive nature and promotion of angiogenesis which supports tumorigenesis and is key for metastasis possible via increased vascular permeability. This is further highlighted by the most dominant pathway with which our biomarker proteins are involved being cell surface interactions at the vascular wall (**Figure 4B**). This indicates a role in facilitating PDAC cell migration through altered or newly formed blood vessels, thereby supporting the dissemination of tumor cells to distant sites.

Finally, the ERK1 and 2 cascades as well as their regulation is another process in which our biomarker proteins are involved. Notably, these extracellular signal kinases are related to several cancers including pancreatic cancers. In fact, KRAS mutations, a key upstream regulator of the ERK pathway, are present in approximately 90-95% of all PDACs. The KRAS-driven activation of the MAPK/ERK pathway results in the phosphorylation of downstream targets, thereby promoting cell cycle progression, survival, and the enhanced metastatic ability characteristic of PDAC cells (36). Though the activation of an aberrant ERK1/2 pathway is known to be activated in PDAC cells, the exact mechanism by which this occurs remains unknown (37, 38). These pathways are integral to PDAC progression and metastasis, and the identified biomarkers may serve as critical indicators of disease status and potential therapeutic targets. Antibody and autoantibody biomarkers in liquid biopsies have been widely identified across various cancers for their potential as non- to minimally invasive diagnostic tools. For example, autoantibodies against HER2 and MUC1 (39, 40) have been reported in breast cancer, while NY-ESO-1 and MAGE-A autoantibodies have been used for diagnostic and prognostic purposes in lung and melanoma cancers (40). Our study extends this approach to PDAC by identifying a novel panel of biomarkers using protein microarrays.

Unlike these single biomarkers identified for other cancers, our panel targets PDAC-specific pathways, such as altered ERK signaling and metabolism, or vascular wall interactions. Interestingly, some overlap exists with autoantibody responses in other cancers, such as MAGEA4 and CEACAM-1, which may reflect shared mechanisms in tumorigenesis. However, our biomarker panel exhibits a higher specificity for PDAC.

Given that our autoantibodies are known to arise in disease through a limited number of mechanisms, our data identify the aberrant expression of specific cancer-testis antigens in individual PDAC patients. Thus, whilst the main objective of this study was to identify a candidate autoantibody-based diagnostic panel for pre-symptomatic and early stage PDAC, the biology of the identified autoantigenic targets also hints at possible paths towards novel precision therapies and cancer vaccines, since the identified cancer-testis antigens appear to be functionally linked to PDAC and are not expressed in other adult somatic tissues, so may represent plausible future targets for autologous immunotherapy.

## Data Availability

The original contributions presented in the study are included in the article/**Supplementary Material**. Raw data will be made available upon reasonable request to interested researchers, direct requests to MO.
