# Supplementary material for: Identification and validation of a novel autoantibody biomarker panel for differential diagnosis of pancreatic ductal adenocarcinoma
Source: Front Immunol. 2025 Jan 30;16:1494446. doi: 10.3389/fimmu.2025.1494446 (PMC11821970; doi:10.3389/fimmu.2025.1494446)
Supplement: Supplementary file 1 [file DataSheet1.docx]

Supplementary Material: Identification of a novel serological pancreatic ductal adenocarcinoma autoantibody biomarker panel with diagnostic and therapeutic implications

**Metoboroghene O. Mowoe^1,5*^, Hisham Ali^2^, Joshua Nqada^2^, Marc Bernon^2^, Karan Gandhi^2^, Sean Burmeister^2^, Urda Kotze^2^, Miriam Kahn^2^, Christo Kloppers^2^, Suba Dharshan^3^, Zafira Azween^3^, Pamela Maimela^1^, Paul Townsend^4^, Eduard Jonas^2^, Jonathan M. Blackburn^1,5*^**

^1^ Department of Integrative Biomedical Sciences, Division of Chemical & Systems Biology, Faculty of Health Sciences, University of Cape Town, Cape Town, South Africa

^2^ Surgical gastroenterology Unit, Division of General Surgery, Groote Schuur Hospital, University of Cape Town, Cape Town, South Africa

^3^ Sengenics Corporation, Floor M, Block A, Plaza Zurich, Damansara Heights, Kuala Lumpur 50490, Malaysia

^4^ Faculty of Health and Medical Sciences, University of Surrey, Guildford, Surrey, United Kingdom

^5^ Institute of Infectious Disease & Molecular Medicine, Faculty of Health Sciences, University of Cape Town, Cape Town, South Africa

*** Correspondence:**Metoboroghene O Mowoe
[m_mowoe@yahoo.co.uk](mailto:m_mowoe@yahoo.co.uk)

Jonathan M Blackburn
jonathan.blackburn@uct.ac.za

# Supplementary Figures and Tables

## Supplementary Figures

##

## Supplementary Figure 1. Fabrication of in-house CT100+ microarrays. a.) Flowchart detailing in-house streptavidin coating of glass susbtrates b.) Position of pins and corresponding microarray spots/probes on substrates for the CT100+ microarray design.

**Supplementary Figure 2. CT100+ and iOme™ anti-c-Myc assay to confirm successful immobilization of in situ purification of biotinylated proteins from lysates on the arrays.** a.) JPEG image of c-Myc array to visualize proteins on the a.) CT100+ and b.) Sengenics iOme ™ arrays. Expression intensities of proteins incubated with anti-c-Myc control on the c.) CT100+ arrays and d.) Sengenics iOme ™ arrays. Each antigen expression intensity was variable because each protein was expressed at different levels within the insect cells and not pre-normalized before array fabrication. For some antigens, signal was reduced possibly because the c-Myc tag was buried within the 3D confirmation of the protein structure. This was corrected at the pre-processing stage downstream.

**Supplementary Figure 3. Western blot analysis of expressed proteins for custom pancreatic ductal adenocarcinoma microarray.** M – Protein ladder; 1 – ALX1; 2 – TPBG; 3- DPPA2; 4- CEACAM1; 5 – GPA33; 6 – XAGE3; 7 – DPPA3; 8 -LIPI; 9- MAGEA4; 10 – SUB1; 11 – SRC

**Supplementary Figure 4. Western blot analysis of expression data of ten of the 11 autoantibodies across ten patient tumour tissue samples.** a.) Barplot of expression intensities of each protein biomarker identified across the ten patient tumour tissue samples, b.) Error bar of log expression intensities of each protein biomarker identified.

**Supplementary Figure 5: ROC curve analysis of validation cohort samples run on the custom pancreatic ductal adenocarcinoma (PDAC) array with the addition of CA19-9.** ROC analysis curve of a.) PDAC versus all diseased and healthy controls; b.) PDAC versus other pancreatic cancers; c.) PDAC versus prostate cancers; and d.) PDAC versus healthy controls.

## Supplementary Tables

| Supplementary Table S1a: Layout of 384-well Plate for the CT100+ Printing Design (Plate 1 of 2) | | | | | |
| --- | --- | --- | --- | --- | --- |
| Well | **Protein** | **Well** | **Protein** | **Well** | **Protein** |
| A1-2,8-9/B1-2,8-9/  C1-2,8-9/D1-2,8-9 | Cy5-biotin-BSA  5 ng/µl | E4,11 | GAGE1 | L5,12 | MART-1/MLANA |
| E1-2,8-9/F1-2,8-9/  G1-2,8-9/H1-2,8-9 | Cy5-biotin-BSA 10 ng/µl | F4,11 | GAGE2A | M5,12 | MICA |
| I1-2,8-9/J1-2,8-9/  K1-2,8-9/L1-2,8-9 | Cy5-biotin-BSA 15 ng/µl | G4,11 | GAGE5 | N5,12 | NLRP4 |
| M1-2,8-9/N1-2,8-9/  O1-2,8-9/P1-2,8-9 | Empty | H4,11 | GAGE6 | O5,12 | NFX2 |
| A3,A10,D4,D11 | bHuIgG 10ng/µl | I4,11 | GAGE6 | P5,12 | NY-CO-45 |
| B3,10 | BAGE2 | J4,11 | GAGE7 | A6,13 | NY ESO 1 |
| C3,10 | BAGE3 | K4,11 | GRWD1 | B6,13 | OIP5 |
| D3,10 | BAGE4 | L4,11 | HORMAD1 | C6,13 | p53 |
| E3,10 | BAGE5 | M4,11 | LDHC | D6,13 | PBK |
| F3,10 | CCDC33 | N4,11 | LEMD1 | E6,13 | RELT |
| G3,10 | CEP290 | O4,11 | LIPI | F6,13 | ROPN1 |
| H3,10 | COL6A1 | P4,11 | MAGEA1 | G6,13 | SGY 1 |
| I3,10 | COX6B2 | A5,12 | MAGEA10 | H6,13 | SILV |
| J3,10 | CSAG2 | B5,12 | MAGEA11 | I6,13 | SPAG9* |
| K3,10 | CT47.11 | C5,12 | MAGEA2 | J6,13 | SPANXA1 |
| L3,10 | CT62 | D5,12 | MAGEA3 | K6,13 | SPANXB1 |
| M3,10 | CTAG2/NY ESO 2 | E5,12 | MAGEA4v2 | L6,13 | SPANXC |
| N3,10 | CXorf48.1 | F5,12 | MAGEA4v3 | M6,13 | SPANXD |
| O3,10 | DDX53 | G5,12 | MAGEA4v4 | N6,13 | SPO11 |
| P3,10 | MMA1 | H5,12 | MAGEA5 | O6,13 | SSX |
| A4,11 | FTHL17 | I5,12 | MAGEB1 | P6,13 | SSX2a |
| B4,11 | Buffer | J5,12 | MAGEB5 |  |  |
| C4,11 | anti-HuIgG | K5,12 | MAGEB6 |  |  |

| Supplementary Table S1b: Layout of 384-well Plate for the CT100+ Printing Design (Plate 2 of 2) | | | | | |
| --- | --- | --- | --- | --- | --- |
| Well | **Protein** | **Well** | **Protein** | **Well** | **Protein** |
| A1,8 | SSX4 | G2,9 | SRCALM1C | M3,10 | TKTL1 Isoform a |
| B1,8 | SYCE1 | H2,9 | CDC25A | N3,10 | SPATS1 Isoform 1 |
| C1,8 | SYCP1 | I2,9 | CREB1 | O3,10 | DPPA2 |
| D1,8 | THEG | J2,9 | CTNNB1 | P3,10 | SOX1 |
| E1,8 | TPTE | K2,9 | p53 S6A | A4,11 | EMPTY |
| F1,8 | TSAG10 | L2,9 | EMPTY | B4,11 | EMPTY |
| G1,8 | TSSK6 | M2,9 | EMPTY | C4,11 | CYPR |
| H1,8 | TYR | N2,9 | EMPTY | D4,11 | EGFR |
| I1,8 | XAGE2 | O2,9 | p53Q136x | E4,11 | ROPNA1 |
| J1,8 | XAGE3a v1 | P2,9 | EMPTY | F4,11 | CEACAM isoform 1 |
| K1,8 | XAGE3 a v2 | A3,10 | EMPTY | G4,11 | POU5F1 |
| L1,8 | ZNF165 | B3,10 | EMPTY | H4,11 | NANOG |
| M1,8 | AKT1 | C3,10 | p53M133T | I4,11 | BORIS BO |
| N1,8 | CDK2 | D3,10 | EMPTY | J4,11 | DPPA4 |
| O1,8 | CDK4 | E3,10 | 5T4/TPBG | K4,11 | DPPA3 |
| P1,8 | CDK7 | F3,10 | XAGE1B | L4,11 | GDF3 |
| A2,9 | FES | G3,10 | SOX2 | M4,11 | CTAG2 |
| B2,9 | FGFR2 | H3,10 | ACVR2A | N4,11 | CAMEL |
| C2,9 | MAPK1 | I3,10 | ACVR2B | O4,11 | NY-ESO 1/ORF2 |
| D2,9 | MAPK3 | J3,10 | ITGB1 | P4,11 | BCCP-myc |
| E2,9 | PRKCZ | K3,10 | MAP9 | A5,12 | Insect cell lysate |
| F2,9 | RAF | L3,10 | PIM1 | B-D5,12/A-D6,13 | Control Buffer |

**Supplementary Table S1c: Layout of 384-well Plate for the Sengenics iOme™ Printing Design**

<https://sengenics.com/resources/protein-list/>

| Supplementary Table S2: CT100+ Assay blocking buffer | | |
| --- | --- | --- |
| Stock | **mL in 500 mL** | **Final concentration (mM)** |
| 1M Hepes, pH 7.5 | 12.5 | 25 |
| Glycerol | 100 | 20% |
| 2.5M KCL | 10 | 50 |
| Triton X-100 | 0.5 | 0.1% |
| 50mM biotin | 0.5 | 0.05 |
| ddH_2_O | 376.5 | - |

| Supplementary Table S3: Array parameters for printing CT100+ microarray | |
| --- | --- |
| Parameter | **Setting** |
| *Environmental* *conditions* |  |
| Temperature | ~ 18 ℃ |
| Humidity | ~45% |
| *Print* *settings* |  |
| Microarray pattern | 8 × 8 spots/grid |
| Total number of source plates | 2 |
| Source order | By column |
| Spacing | 562 µm |
| Max. stamps per ink | 1 |
| Stamps per spot | 1 |
| Stamp time | 0 ms |
| Inking time | 500 ms |
| Print depth adjustment | 150 µm |
| Number of touch offs | 0 |
| *Pin wash setting* |  |
| Water washes | 60 s |
| Ethanol wash | 10 s |
| Drying | 10 s |
| Pause | 1 s |

| Supplementary Table S4: Area under the Receiver operating characteristics Curve, Cut-offs, Specificity, Sensitivity of Autoantigens Required for Pancreatic Ductal Adenocarcinoma Diagnosis against diseased controls | | | | |
| --- | --- | --- | --- | --- |
| Autoantigen | **AUC** | **Cut-off (log expression)** | **Specificity** | **Sensitivity** |
| MAGEA4 | 0.709 | 10.694 | 0.795 | 0.579 |
| SRC | 0.704 | 9.845 | 0.800 | 0.526 |
| 5T4.TPBG | 0.675 | 9.571 | 0.800 | 0.579 |
| SYCE1 | 0.669 | 10.475 | 0.720 | 0.631 |
| GRWD1 | 0.667 | 11.134 | 0.820 | 0.526 |
| CEACAM 1 | 0.654 | 9.269 | 0.700 | 0.579 |
| XAGE 3 | 0.653 | 10.381 | 0.620 | 0.736 |
| DPPA3 | 0.652 | 9.175 | 0.800 | 0.526 |
| LIPI | 0.649 | 10.273 | 0.760 | 0.526 |
| DPPA2 | 0.648 | 8.581 | 0.700 | 0.579 |

| Supplementary Table S5: Results of Western blot analysis of proteins in the custom PDAC array | | | | |
| --- | --- | --- | --- | --- |
| Lane | **Gene Symbol** | **Expected MW (kDa)** | **Observed MW (kDa)** | **Uniprot** |
| M | Biotinylated Protein Ladder | -- | -- | -- |
| 1 | CEACAM1 | 63.3 | 100 | P13688 |
| 2 | DPPA2 | 46.6 | 51.4 | Q7Z7J5 |
| 3 | DPPA3 | 32.6 | 32.3 | CT119 |
| 4 | MAGEA4 | 47.7 | 54.9 | P43358 |
| 5 | SRC | 75.8 | 65.4 | CT83 |
| 6 | TPBG | 59.0 | 67.5 | Q13641 |
| 7 | XAGE3 | 37.6 | 33.1 | CT71 |
| 8 | ALX1 | 49.7 | 52.7 | Q15699 |
| 9 | GPA33 | 48.4 | 58.2 | Q99795 |
| 10 | LIPI | 68.1 | 63.3 | Q6XZB0 |
| 11 | SUB1 | 27.1 | 30.6 | P53999 |
